# Systemic Ketone Replacement Does Not Improve Survival or Cancer Cachexia in Mice With Lung Cancer

**DOI:** 10.3389/fonc.2022.903157

**Published:** 2022-06-03

**Authors:** Henning Tim Langer, Shakti Ramsamooj, Roger J. Liang, Rahul Grover, Seo-Kyoung Hwang, Marcus DaSilva Goncalves

**Affiliations:** ^1^ Division of Endocrinology, Weill Department of Medicine, Weill Cornell Medicine, New York, NY, United States; ^2^ Meyer Cancer Center, Weill Department of Medicine, Weill Cornell Medicine, New York, NY, United States

**Keywords:** ketogenic diet (KD), ketone ester supplementation, cachexia, lung cancer, PPAR alpha, beta hydroxybutyrate

## Abstract

Cachexia is a debilitating comorbidity affecting many lung cancer patients. We have previously found that cachectic mice with lung cancer have reduced serum ketone body levels due to low PPARα activity in the liver. Restoring hepatic PPARα activity with fenofibrate increased circulating ketones and delayed muscle and white adipose tissue wasting. We hypothesized that the loss of circulating ketones plays a pathophysiologic role in cachexia and performed two dietary intervention studies to test this hypothesis. In the first study, male and female mice were randomized to consume either a very low carbohydrate, ketogenic diet (KD) or normal chow (NC) after undergoing tumor induction. The KD successfully restored serum ketone levels and decreased blood glucose in cachectic mice but did not improve body weight maintenance or survival. In fact, there was a trend for the KD to worsen survival in male but not in female mice. In the second study, we compounded a ketone ester supplement into the NC diet (KE) and randomized tumor-bearing mice to KE or NC after tumor induction. We confirmed that KE was able to acutely and chronically increase ketone body abundance in the serum compared to NC. However, the restoration of ketones in the circulation was not able to improve body weight maintenance or survival in male or female mice with lung cancer. Finally, we investigated PPARα activity in the liver of mice fed KE and NC and found that animals fed a ketone ester supplement showed a significant increase in mRNA expression of several PPARα targets. These data negate our initial hypothesis and suggest that restoring ketone body availability in the circulation of mice with lung cancer does not alter cachexia development or improve survival, despite increasing hepatic PPARα activity.

## Introduction

Lung cancer is one of the leading causes of death in the United States. Patients with lung cancer have a high prevalence of cachexia, which independently predicts the length of survival, response to anti-cancer therapy, and quality of life (1–4). Cancer cachexia is a systemic metabolic syndrome characterized by the wasting of skeletal muscle and adipose tissue. It is thought to arise from tumor-secreted factors which alter host tissue metabolism and activate catabolic signaling. In addition to the skeletal muscle and adipose tissue, we and others have described the dramatic effects that occur in the liver during cachexia (5–8).

The liver is a critical mediator of metabolic homeostasis during health and disease. The liver is centrally involved in regulating systemic carbohydrate metabolism by both storing and releasing glucose in times of energy surplus and deficit, respectively. The liver also regulates systemic fatty acid metabolism and is the primary site of ketone body production. Both glucose and ketone bodies alter skeletal muscle and adipose tissue metabolism. For example, ketone bodies such as beta hydroxybutyrate decrease amino acid degradation and promote protein synthesis in human skeletal muscle (9). Therefore, diseases that alter hepatic ketone metabolism can have a broad impact on skeletal muscle and other peripheral organs.

Ketogenesis and fatty acid oxidation are primarily regulated by peroxisome proliferator-activated receptor (PPAR)-α, a nuclear receptor activated by several endogenous and dietary polyunsaturated fatty acids (10). We and others have previously found that mice with cachexia have reductions in hepatic fatty acid oxidation and ketone metabolism, arising from inactive PPARα (5, 6). When PPARα activity is therapeutically induced with fenofibrate, a clinically approved PPARα agonist, the deleterious changes in hepatic fatty acid metabolism are reversed, blood ketone levels rise, and cachexia is prevented in mice with lung cancer (5). Based on these data, we hypothesized that fenofibrate preserves peripheral tissue mass and metabolism during cachexia by increasing serum ketone levels. To test this hypothesis, we conducted two prospective, randomized, nutritional intervention trials that directly and indirectly increased ketone body availability, and analyzed the subsequent changes in body weight, survival, and relevant markers of cachexia in mice with lung cancer.

## Materials and Methods

### Cachexia Model and Lung Tumor Induction

Kras^G12D/+^;Lkb1^f/f^ mice have been previously described (11). Mice were housed in a 12-h light/dark cycle and 22°C ambient temperature and had free access to normal chow (PicoLab Rodent 20 5053; Lab Diet) and drinking water. Tumors were induced in adult (12- to 20-wk-old) male and female mice *via* intranasal administration of 75 μL of PBS containing 1 mM CaCl_2_ and 2.5 × 10 (7) pfu of Adenovirus CMV-Cre (Ad5CMV-Cre) purchased from the University of Iowa Gene Transfer Vector Core (Iowa City, IA). A detailed description of the cachectic phenotype in these mice, its time-course, and its severity have been published previously (5). Briefly, the animals develop a cachectic phenotype that comprises progressive atrophy in type II myofibers, reduced muscle and total body mass, and worsened spontaneous and forced activity about 6 weeks after tumor induction. The duration of the cohorts are 9-12 weeks and mice are euthanized if they reach the following humane endpoints: >30% loss from peak body weight or poor body composition score (<2). All animal studies were approved and maintained as approved by the Institutional Animal Care and Use Committee (IACUC) of Weill Cornell Medicine under protocol number 2013-0116.

### Interventional Study Design

#### Intervention 1

Mice (15 male and 8 female) were induced with Ad5CMV-Cre. Four weeks after induction, mice were stratified by sex and randomized to continue normal chow or start a very low carbohydrate (ketogenic) diet (BioServ S3666) for the duration of the study. Animals were assessed and weighed weekly. Animals were euthanized if they exceeded a weight loss of 30% from their peak weight during the study or if they developed a poor body composition score (<2). All remaining animals were euthanized at 12 weeks after tumor induction if they did not yet meet euthanasia criteria.

A pilot study was performed on mice (9 male and 8 female) that did not undergo tumor induction to test the safety and efficacy of the ketone ester diet. A subset of the mice (6 male and 5 female) was fed normal chow infused with 10% (v/w) ketone ester (1,3-butanediol-acetoacetate provided by Disruptive Enterprises, LLC). The ketone ester diet was compounded, pelleted, and irradiated by LabDiet (St. Louis, MO). The remaining mice continued the standard normal chow diet. After 3 days of acclimation, blood was sampled from the tail vein every 4 hours for 20 hours to measure beta hydroxybutyrate levels using a point-of-care ketone meter (Precision Xtra). The mice were then euthanized.

#### Intervention 2

Mice (11 male and 10 female) were induced with Ad5CMV-Cre. Four weeks after tumor induction, mice were randomized to continue normal chow or start the ketone ester diet. The remainder of the study was the same as Intervention 1.

### Tissue Collection Protocol

Prior to euthanasia, mice were food-deprived for 3 h and then glucose was measured from tail vein blood using a handheld point-of-care glucose meter (OneTouch). Euthanasia was performed using CO_2_ asphyxiation. Following euthanasia, whole blood was collected *via* cardiac puncture and placed into serum separator tubes on ice. Subsequently, the whole liver was removed, weighed, and frozen in liquid nitrogen. The white adipose tissue, lungs, spleen and skeletal muscles were dissected, weighed, and flash-frozen in liquid nitrogen or fixated in paraformaldehyde. All tissues were subsequently stored at −80°C until further processing.

### Serum Metabolites and Corticosterone

Blood was centrifuged (10,000 × g for 10 min at 4°C), and serum was stored at −20°C. Serum β-hydroxybutyrate, Triglycerides (Stanbio Laboratory), non-esterified fatty acids (Wako Life Sciences), were determined using commercially available kits. Serum corticosterone was quantified by ELISA (ALPCO Diagnostics). The number of samples per cohort that we were able to include in our serum analysis varied on the basis of available blood volume and sensitivity of the kits.

### RT-qPCR

Total RNA was extracted from liver tissue (30–50 mg) using TRIzol (Invitrogen) and a tissue homogenizer (Wuhan Servicebio technology) at 70 hz for two rounds of 70 s. After confirming purity, the samples underwent reverse transcription using VILO Master Mix (Thermo Fisher). Samples were stored at -80°C before the qPCR step was conducted on the next day. Transcripts were amplified using Applied Biosystems TaqMan Gene Expression Assays (Thermo Fisher) with the following primers: Acox1 (Mm01246834_m1), Actb (Mm00607939_s1), Bdh1 (Mm00558330_m1), Ehhadh (Mm00619685_m1), Hmgcs2 (Mm00550050_m1), Rer1 (Mm00471276_m1), Rplp0 (Mm00725448_s1), Rpl7l1 (Mm00786031_s1). The mRNA expression levels of the genes of interest were normalized to the arithmetic mean of the four housekeeping-genes and calculated according to the delta-delta ct method (12). All animals included in the analysis were tumor bearing and displayed a weight loss of >10% from peak weight.

### Statistics

Statistical analysis was carried out using Prism 9.3.1 (GraphPad Software). Individual data points are provided for every graph and assay with exception of time-course graphs (survival curves, acute ketone ester experiment). The mean ± SEM is displayed in addition to the individual data. The statistical test applied was dependent on the analysis performed and is indicated in each legend. Unless otherwise stated, a two-way ANOVA was performed to detect main effects for the dietary intervention, sex differences and any potential interactions between diet and sex. Sidak’s multiple comparison test was run *post-hoc* to investigate individual differences in the diet response for each sex. The time course of the acute ketone ester experiment was investigated using a repeated measures two-way ANOVA and Holm-Sidak’s multiple comparison test. Survival curves were compared by a Log-rank (Mantel-Cox) test. Samples from male and female mice from the ketone ester cohorts were clustered for RT-qPCR analysis and group differences assessed by an unpaired t-test. A p-value of <0.05 was deemed as statistically significant, values between 0.05 and 0.1 are referred to as trends.

## Results

### A Ketogenic Diet Increases Ketone Bodies and Decreases Glucose Levels in Cachectic Mice With Lung Cancer

**Figure 1:**
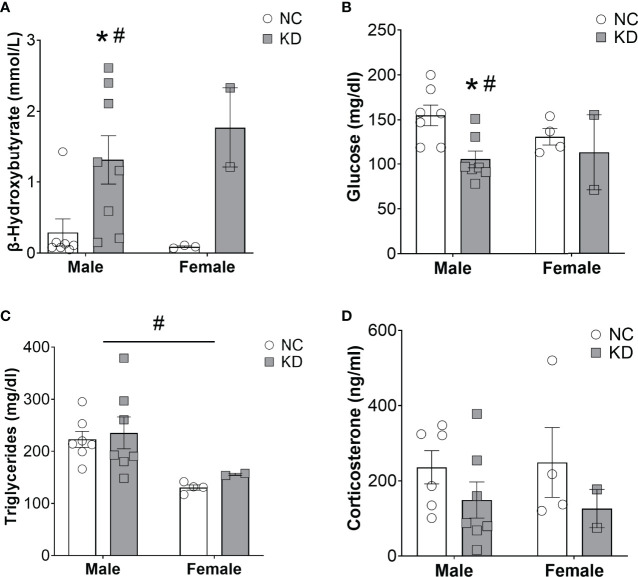
** **A ketogenic diet increases ketone bodies and decreases glucose levels in cachectic mice. Serum beta hydroxybutyrate **(A)**, glucose **(B)**, triglycerides **(C)**, and corticosterone **(D)** levels from male and female mice consuming a normal chow diet (NC) or a ketogenic diet (KD). Bars denote mean +/- SEM. ^#^ denotes a significant main effect (p < 0.05) of diet or sex of the animals *via* a two-way ANOVA, while a * denotes a p-value of < 0.05 for *post-hoc* analysis comparing NC *vs* KD within each sex. We omitted the *post-hoc* serum analysis for the female cohorts based on the lack of an appropriate sample size in the KD group. Out of originally four animals in the cohort, two died prematurely leaving us unable to collect sufficient blood for serum analysis.

If ketones are essential to preventing the loss of skeletal muscle and adipose tissue mass during cachexia, then reactivating ketogenesis with a dietary intervention should prevent cachexia. This theory was tested using a high-fat, moderate-protein, very low carbohydrate (ketogenic) diet, which is known to initiate ketone body production in the liver (13). The diet was started four weeks after tumor induction to allow for normal tumor development. At this time, mice (15 male and 8 female) were stratified by sex and randomized to continue normal chow (NC) or start a very low carbohydrate (ketogenic) diet (KD) until they met endpoint criteria (30% weight loss, a body composition score <2, or survival at 12 weeks after induction). Beta hydroxybutyrate levels in the serum of male mice on KD were elevated to 1.3 mmol/l compared to 0.3 mmol/l in male mice on a standard chow diet. Similarly, female mice on a ketogenic diet had mean beta hydroxybutyrate levels of 1.8 mmol/l compared to 0.1 mmol/l in female mice on the control diet. These differences resulted in a significant main effect for the diet intervention (p<0.01), and significant *post-hoc* differences for both sexes compared to the control groups (p<0.05) (**Figure 1A**). Glucose levels were decreased by 47% in male mice on a ketogenic diet and by 16% in female mice on a ketogenic diet compared to the control groups, resulting in a significant main effect for the dietary intervention (p<0.05) and a *post-hoc* difference between the male cohorts (p<0.05) (**Figure 1B**). There was a main effect for a sex indicating lower levels of circulating triglycerides in female mice (p<0.01) but no effect of the diet (p=0.52) (**Figure 1C**). The ketogenic diet reduced circulating corticosterone levels by 87 ng/ml in male and by 123 ng/ml in female mice, but this effect failed to reach statistical significance (p=0.15) (**Figure 1D**).

### Increased Ketone Availability and Decreased Glucose Do Not Improve Survival in Cachectic Mice With Lung Cancer

**Figure 2:**
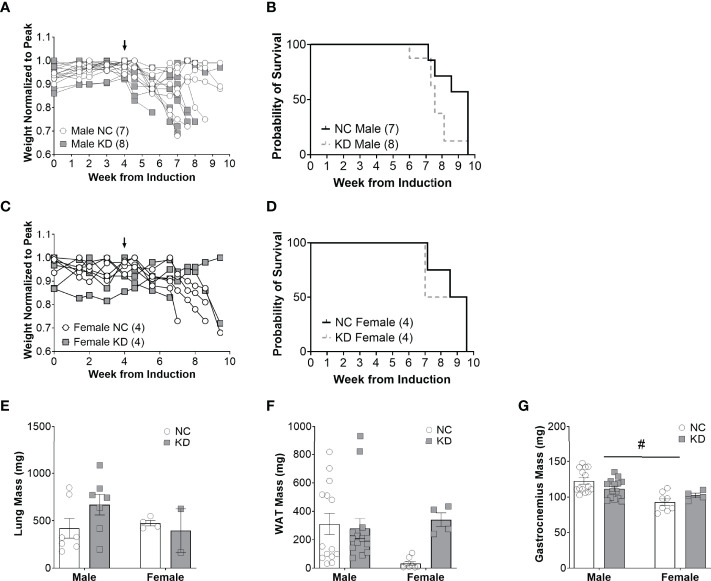
Increased ketone availability and decreased glucose do not improve survival in cachectic mice with lung cancer. Individual body weight changes of male **(A)** and female **(C)** mice on a normal chow (NC) or ketogenic diet (KD) normalized to peak body weight over the course of the study. The corresponding overall survival curves **(B, D)**, lung mass **(E)**, white adipose tissue (WAT) mass **(F)**, and gastrocnemius mass **(G)** are shown. For the WAT and gastrocnemius, the individual data points on the graph include biological replicates in the form of bilateral tissues from the same animal. There was no significant main effect for the diet on survival but a trend for animals on the KD to live a shorter duration (p=0.07). Statistical analysis for E, F, and G was performed *via* two-way ANOVA. ^#^ denotes a significant main effect (p < 0.05) of sex of the animals. Sample size is indicated in parentheses in the legends of **(A–D)**. The arrows indicate the start of the dietary intervention. We omitted the *post-hoc* analysis for tissues of the female cohorts based on the lack of an appropriate sample size in the KD group. Out of originally four animals in the cohort, two died prematurely leaving us unable to collect the tissue.

Despite the changes in serum beta hydroxybutyrate and blood glucose, the median survival of male mice on a ketogenic diet was two weeks shorter than mice on a standard diet (KD: 7.6 weeks, NC: 9.6 weeks, p=0.07) (**Figure 2B**). Median survival in female mice on a ketogenic diet was 8.3 weeks compared to 9.1 weeks for mice on the control diet (p=0.8) (**Figure 2D**). Lung mass, a surrogate for tumor burden, was 59% greater in male animals fed a ketogenic diet compared to the normal chow group, but there were no statistically significant main effects for diet (p=0.58), sex (p=0.3), an interaction between diet and sex (p=0.12), or *post-hoc* for the male mice specifically (p=0.17) (**Figure 2E**). White adipose tissue showed no main effect for the diet (p=0.11) or sex (p=0.2) but a trend toward an interaction between diet and sex (p=0.05), and a trend toward more white adipose tissue in female mice fed a ketogenic diet (p=0.07) (**Figure 2F**). For the gastrocnemius, there was no main effect for the diet (p=0.89) but a significant effect for sex (p<0.001), a trend for an interaction effect between diet and sex (p=0.05), and a *post-hoc* trend toward a significant decrease in muscle mass for male mice fed a ketogenic diet (p=0.09) (**Figure 2G**).

### Ketone Ester Supplementation Acutely and Chronically Elevates Ketone Body Levels

**Figure 3:**
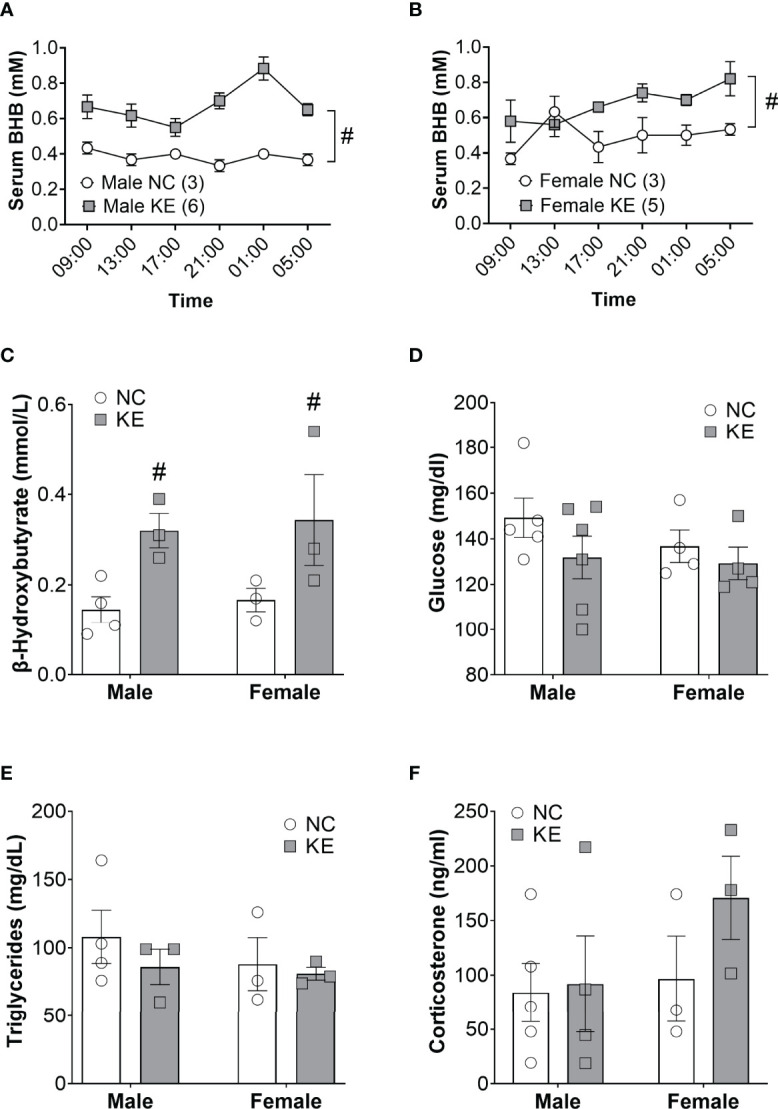
Ketone ester supplementation acutely and chronically elevates ketone body levels. Non-tumor bearing mice were fed normal chow (NC) or NC supplemented with a ketone ester (KE). The resulting serum beta hydroxybutyrate levels over the course of 20 hours are shown for male **(A)** and female **(B)** mice. Tumor-bearing mice were fed NC or KE and the resulting serum beta hydroxybutyrate **(C)**, glucose **(D)**, triglycerides **(E)**, and corticosterone **(F)** levels are shown. Statistical analysis for A and B were performed using a two-way repeated measures ANOVA (p < 0.001 for male and p<0.05 for female), with *post-hoc* testing showing that serum BHB levels were significantly elevated at every time point in KE males (p<0.05) but not females. For **(C–F)** statistical testing was performed *via* two-way ANOVA. ^#^ denotes a significant main effect (p < 0.05) of the diet of the animals. Sample size is indicated in parentheses in the legends of **(A, B)**.

Ketogenic diets modulate a broad array of metabolic hormones and metabolites in addition to serum ketones. To directly assess the role of the ketone body in cachexia, we fed mice a ketone ester supplement (1,3-butanediol-acetoacetate), which is metabolized to beta hydroxybutyrate and acetoacetate by the liver (14). Ketone ester supplements increase the blood ketone levels without global distortions in systemic metabolism (15, 16). To confirm the efficacy and safety of feeding mice a ketone ester supplement, we performed a short pilot study in non-tumor bearing mice to measure the abundance of beta hydroxybutyrate in the circulation over a 24-hour period. In male mice, we found a significant main effect for the diet to elevate beta hydroxybutyrate levels (p<0.001). *Post-hoc* analysis determined that beta hydroxybutyrate levels were elevated with the ketone ester diet for every single time-point (p<0.05) (**Figure 3A**). Similarly, in female wildtype mice beta hydroxybutyrate levels were significantly increased for the ketone ester diet group (p<0.05) while *post-hoc* analysis did not reveal significant differences for individual time points (**Figure 3B**). In a 2-week pilot study using non-tumor bearing mice, the ketone ester diet had no deleterious effects on food intake, body weight, or spontaneous activity (**Supplementary Figure**) so we proceeded to perform an intervention study in tumor bearing mice.

Like the ketogenic diet intervention study, we induced mice (11 male and 10 female) with Ad5CMV-Cre and then, 4 weeks later, stratified by sex and randomized to continue normal chow or start the ketone ester diet until endpoint criteria. Beta hydroxybutyrate levels in the serum of tumor bearing mice showed a significant main effect for the ketone ester diet to elevate circulating ketones (p<0.01) (**Figure 3C**). Serum glucose levels were not significantly impacted by the ketone ester supplementation (p=0.18) (**Figure 3D**). We observed no significant main effects of the diet or the sex of the animals for circulating triglyceride (**Figure 3E**) or corticosterone levels (**Figure 3F**).

### Increased Ketone Availability *via* Dietary Supplementation Does Not Improve Survival in Mice With Lung Cancer

**Figure 4:**
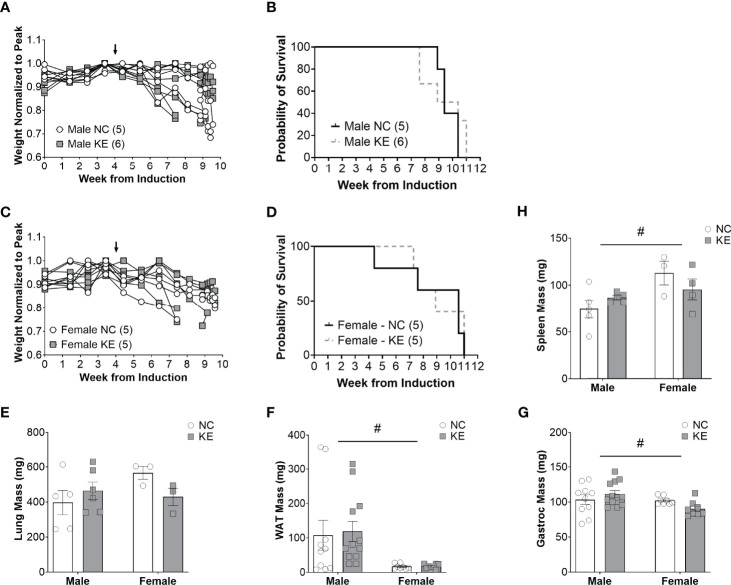
Increased ketone ester availability does not improve survival in mice with lung cancer. Individual body weight changes of male **(A)** and female **(C)** mice consuming a normal chow (NC) or NC supplemented with a ketone ester (KE) normalized to peak body weight over the course of the study. The corresponding overall survival curves **(B, D)**, lung mass **(E)**, white adipose tissue (WAT) mass **(F)**, gastrocnemius **(G)**, and spleen mass **(H)** are shown. For the WAT and gastrocnemius, the individual data points on the graph include biological replicates in the form of bilateral tissues from the same animal. There was no significant main effect for the diet on survival. Statistical analysis for **(E-G)** was performed *via* two-way ANOVA. ^#^ denotes a significant main effect (p < 0.05) of sex of the animals. Sample size is indicated in parentheses in the legends of **(A-D)**. The arrows indicate the start of the dietary intervention.

Despite the combination of a balanced diet with increased ketone availability, male mice on a ketone ester supplemented diet lived just as long (a median of 9.7 weeks) as mice on a standard diet (a median of 9.4 weeks) (p=0.62) (**Figure 4B**). Similarly, female mice on a ketone ester supplemented diet lived a median of 8.9 weeks after induction of the virus, while animals on the control diet lived a median of 10.6 weeks (p=0.77) (**Figure 4D**). There was no main effect for the diet (p=0.58), sex (p=0.3), or the interaction between diet and sex (p=0.12) for lung weight (**Figure 4E**). For white adipose tissue we found a significant difference for sex of the animals (p<0.01) with white adipose tissue mass being on average about 82% lower in female compared to male mice (**Figure 4F**). There was no effect of the diet (p=0.84) or an interaction between diet and sex (p=0.86) for white adipose tissue. While there was no main effect of the diet (p=0.72) on gastrocnemius mass, there was a main effect for sex differences (p<0.05) and a trend toward an interaction effect between the sex and the diet (p=0.06), with mass tending to increase in male- and decrease in female mice (**Figure 4G**). There was a significant sex difference for spleen mass (p<0.05) but no effect of the diet (p=0.79) and no interaction effect (p=0.13) (**Figure 4H**).

### Ketone Ester Supplementation Activates Hepatic PPARα

**Figure 5:**
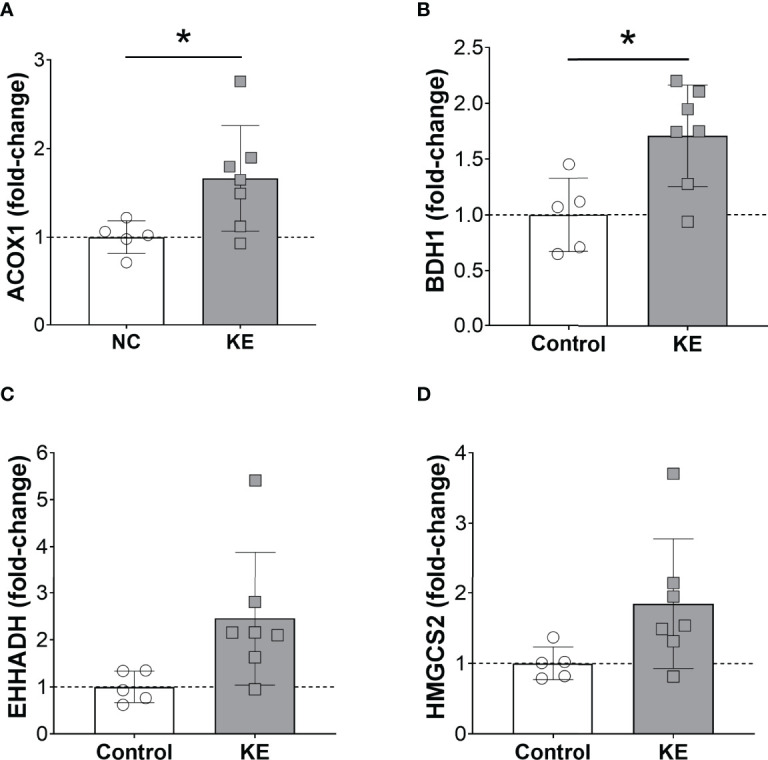
Ketone ester supplementation activates hepatic PPARα. Hepatic mRNA expression of the PPARα target genes *Acox1* **(A)**, *Bdh1* **(B)**, *Ehhadh* **(C)**, and *Hmgcs2* **(D)** in mice fed a normal chow diet (NC) or NC supplemented with a ketone ester supplement (KE). Male and female tissues were combined as no sex-dependent effects were observed. Statistical comparisons between NC and KE were made using a student’s t-test and * denotes a significant difference (p<0.05).

We examined PPARα activity in the liver of mice fed normal chow and the ketone ester diet. We found that the ketone ester supplementation increased the mRNA expression of hepatic PPARα targets. *Acox1* mRNA expression increased by 66% in the liver of mice on the ketone ester diet compared to the control diet (p<0.05) (**Figure 5A**). Similarly, *Bdh1* expression increased by 70% in mice fed a ketone ester diet (p<0.05) (**Figure 5B**). In line with these observations, we observed trends for increased *Ehhadh* and *Hmgcs2* expression. *Ehhadh* increased to 2.5-fold (p=0.05) (**Figure 5C**) and *Hmgcs2* by 85% (p=0.07) (**Figure 5D**) in mice on a ketone ester diet compared to a standard chow diet.

## Discussion

In this study, we used two dietary interventions to test the hypothesis that circulating ketones protect peripheral tissue mass during cachexia. Both the ketogenic diet and ketone ester supplement significantly increased blood ketones in mice with lung cancer, however this increase did not prevent or delay the onset of cachexia. Furthermore, there was no preservation of skeletal muscle or adipose tissue mass, and overall survival did not change. These data clearly negate our hypothesis.

The ketogenic diet has been suggested as a treatment for a wide range of medical conditions, including migraines (17), epilepsy (18), multiple sclerosis (19), Alzheimer’s disease (20), diabetes (21), aging (22), cancer (23), and cancer associated cachexia (24). One of the primary mechanisms underlying the purported benefits of the ketogenic diet is the switch from glucose as a primary fuel to fatty acids and ketone bodies. In support of this concept, we previously reported decreased levels of beta hydroxybutyrate in mice with lung cancer induced cachexia, and significant anti-cachectic benefits when ketogenesis was restored with the PPARα agonist, fenofibrate (5).

In this study, we anticipated that the ketogenic diet would activate PPARα, restore ketogenesis, and protect against cachexia; however, this was not the case. While animals on a ketogenic diet have substantially higher circulating levels of ketones, this response did not preserve skeletal muscle mass or body weight. Furthermore, we observed several worrisome trends when mice with lung cancer were fed a ketogenic diet. Male mice tended to have increased tumor burden, decreased skeletal muscle and adipose tissue mass, and worse survival. These effects were not observed in the mice fed the ketone ester supplement, which suggests that the ketone body itself is not directly involved in these responses. There are numerous differences between the ketogenic diet and normal chow that could be contributing to these effects, including the low carbohydrate and fiber content, the moderate protein restriction, and large amount of dietary lipids. Bhatt *et al.* previously described how loss of *Lkb1* makes lung tumors dependent on fatty acid oxidation, so we speculate that the excess dietary lipids are a major contributor to the worsened survival (25).

In an attempt to avoid large changes in dietary macronutrient content, we created a well-balanced dietary formulation that contains exogenous ketones in the form of a ketone ester. This diet increased the availability of ketones in the circulation of mice with and without lung cancer. Nevertheless, the ester had no effect on survival, body weight, nor skeletal muscle and adipose tissue mass when it was fed to tumor-bearing mice. These data suggest that “ketone replacement therapy” using a ketone ester supplement or ketogenic diet is not a useful strategy to prevent cachexia in mice with lung cancer.

Previously, we found that mice with lung cancer induced cachexia have decreased PPARα activity in the liver (5). Restoring hepatic PPARα with fenofibrate was associated with an improved disease course and preserved muscle and adipose tissue mass. To investigate whether PPARα activity plays a pathophysiologic role in cancer cachexia in this model, we examined PPARα activity in the liver of mice fed normal chow and the ketone ester diet. Interestingly, we found that the ester-fed mice had increased expression levels of PPARα gene targets in the liver. To our knowledge, this is the first study to show this effect and it remains unclear how the ester amplifies PPARα activity. The ester may increase the total abundance of PPARα by stimulating short-chain fatty acid receptors such as GPR41/FFA3 (26), GPR43/FFA2 (27) or other G protein-coupled receptors like GPR109A/HCA2 (28), as has been shown in neuronal tissue (29). Beta hydroxybutyrate can also activate AMPK in the livers of rats and AMPK is known to be an upstream regulator of PPARα in various tissues (30–35). Nevertheless, the activation of PPARα was not associated with improved tissue preservation or survival in our model. These data suggest that the restoration of PPARα in the liver was not driving the beneficial effects of fenofibrate in our prior study (5).

Our results are in contrast to other reports that describe improvements in weight and survival in mouse models of cancer fed ketogenic diets (36–38) and ketone ester supplements (39). These disparate effects may be due to the inherent differences in tumor location and molecular drivers of tumor growth in each pre-clinical model. In this study, we used a genetic model of lung cancer driven by an activating mutation in *Kras* and loss of the tumor suppressor gene, *Lkb1* (*Stk11*). This combination of genetic alterations enhances tumor fatty acid oxidation and, in some instances, upregulates ketolytic enzymes (25, 40, 41). In this setting, the ketogenic diet may enhance tumor progression by stimulating tumor growth and create deleterious changes to the tumor microenvironment (42). An additional reason for the discrepancies to other studies could be the composition of our diet and dosage of the ketone esters, as other studies with successful outcomes have used a higher dietary content of ketones in the diet (39).

One limitation of our study is the small sample size of the female mice in the ketogenic diet cohort, which limits our ability to detect sex-specific effects. The data were separated by sex to highlight the known differences in muscle mass and distinct changes in white adipose tissue that occur in response to diet. To that end, we observed that female mice with lung cancer maintained more white adipose tissue on a ketogenic diet than male mice; however, this change had no positive effect on survival nor body weight. These observations add to the recent and important appreciation for sex differences in the field of cancer cachexia (43–47). Another limitation is the lack of a functional analysis of the skeletal muscle tissue, which would have allowed us to investigate whether there had been changes in performance that were independent from changes in muscle mass.

In summary, increasing ketone body availability and hepatic PPARα activity through a ketogenic diet or ketone ester supplementation in mice with lung cancer did not increase survival, nor did it improve the maintenance of body weight and muscle mass. Future research needs to delineate the primary mechanism through which tissue wasting is driven in this model to improve the design of adequate therapeutical interventions.

## Data Availability Statement

The raw data supporting the conclusions of this article will be made available by the authors, without undue reservation.

## Ethics Statement

The animal study was reviewed and approved by Weill Cornell Institutional Animal Care and Use Committee.

## Author Contributions

HTL performed sample analyses, data analyses and drafted the manuscript. SR, RL, RG, and S-KH performed the animal studies and part of the sample analyses. MG conceptualized the project, performed sample analyses, and helped draft the manuscript. All authors contributed to the article and approved the submitted version.

## Funding

This work was supported by a grant from the Lung Cancer Research Foundation (MG), NIH K08 CA230318 (MG), and the 2020 AACR-The Mark Foundation for Cancer Research “Science of the Patient” (SOP) Grants, Grant Number 20-60-51-GONC.

## Conflict of Interest

MG has received research support from Pfizer, Inc. and is a co-founder and shareholder in Faeth Therapeutics, which is developing treatments for cancer outside the scope of the current work. MG was paid to attend and participate in an advisory board meeting hosted by Disruptive Enterprises, LLC in 2019.

The remaining authors declare that the research was conducted in the absence of any commercial or financial relationships that could be construed as a potential conflict of interest.

## Publisher’s Note

All claims expressed in this article are solely those of the authors and do not necessarily represent those of their affiliated organizations, or those of the publisher, the editors and the reviewers. Any product that may be evaluated in this article, or claim that may be made by its manufacturer, is not guaranteed or endorsed by the publisher.
