# Supplementary figures and images for: Systemic Ketone Replacement Does Not Improve Survival or Cancer Cachexia in Mice With Lung Cancer
Source: Front Oncol. 2022 Jun 3;12:903157. doi: 10.3389/fonc.2022.903157 (PMC9203842; doi:10.3389/fonc.2022.903157)

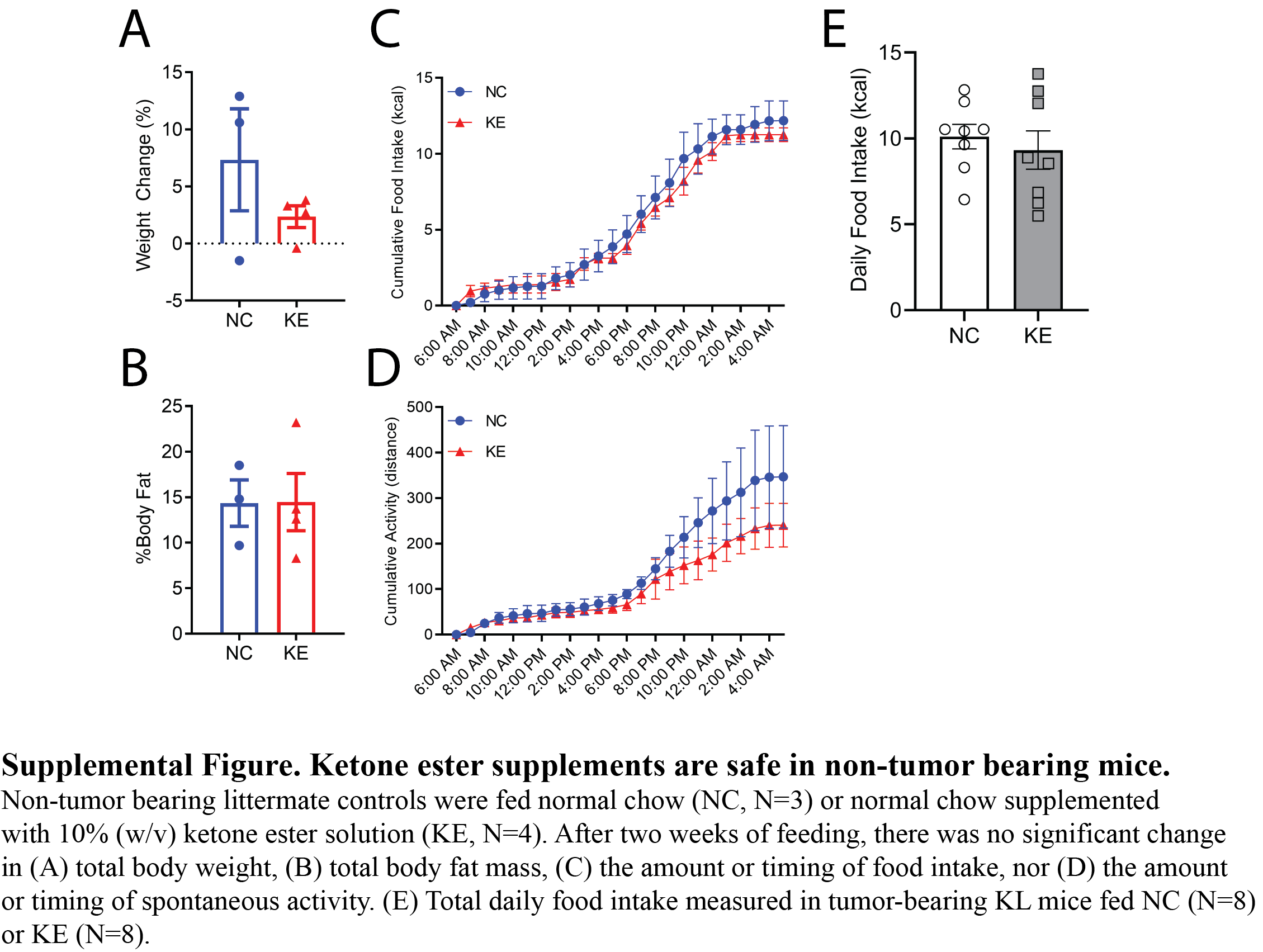

Supplement: Supplementary file 1 [file Image_1.png]
